# 

**Published:** 1860-08

**Authors:** 


Published Monthly, at $3 per Annum in Advance.

### ATLANTA

### MEDICAL AND SURGICAL

### JOURNAL.

EDITED BY

**JOSEPH P. LOGAN, M. D.,**

PROFESSOR OF PHYSIOLOGY AND DISEASES OF WOMEN AND CHILDREN IN THE ATLANTA MEDICAL COLLEGE.

AND

**W. F. WESTMORELAND, M. D.,**

PROFESSOR OF THE PRINCIPLES AND PRACTICE OF SURGERY IN THE ATLANTA MEDICAL COLLEGE.

**Pax et scientia, sed veritas sine timore.**

VOL. V. AUGUST, 1860. No. XII.

ATLANTA, GA:
ATLANTA INTELLIGENCER PRINT.
1860.

Each Number contains Sixty-Four Pages.

